# 

**Published:** 1914-11-21

**Authors:** 


### CONTENTS.

| | PAGE |
|---|---|
| **The War and Hospitals for Nervous Diseases** | 163 |
| **The Institutional Problem of Mental Defectives** | 164 |
| **Hospital and Institutional News ... ... ...** | 165 |

The First Medical Victoria Cross—The Meaning of Medical Valour—The Queen's Visit to the American Women's War Hospital—The Five Points of a Successful Appeal—A Dispensing Disaster at Bethlem Hospital—A Dispensing Reform in Mental Hospitals—The New Sanatorium for Women—Farm Labour for Consumptives—The Crux of the After-care Problem—The Wounded "Keep the Hospitals Going"—State Aid for the Fine Chemical Trade—Tribute to a Hospital Secretary—Essex Doctors' Gift to the War Office—The War and Hospital Saturday—A Panel Doctor "Fined"—Discipline in Convalescent Homes—A Death at a Board Meeting—Medical Men on Public Bodies—Belgian Soldiers as Hospital Collectors—Welsh Dissatisfaction with Sanatorium Benefit—First Step in the Anti-syphilis Campaign—How to Help the Ambulance Service—Boots for the Belgian Wounded—This Week's Drug Market.

| | PAGE |
|---|---|
| **With a British Field Ambulance in Retreat...** | 171 |

By an Army Medical Officer at the Front.

[See over.]

### CONTENTS—(*continued*).

| | PAGE |
|---|---|
| **The New Tuberculosis Hospital at Lubeck ...** Latest Type of German Tuberculosis Pavilions. | 172 |
| **St. Bartholomew's Hospital and the Wounded** Two Months' Experience of War Work. | 173 |
| **The Iodine Method of Skin Sterilisation ...** Its Value against Septic Infection. | 174 |
| **Hospitals and the War ... ... ... ...** | 175 |
| **Work for the Wounded of the Allied Armies** Nelson Hospital, Wimbledon—Bath—Brighton: 2nd Eastern General Hospital Enlarged—Sunderland: The Second Convoy—Coventry. | |
| **British Wounded in a French Hospital at St. Malo ... ... ... ... ... ...** | 176 |
| **Accommodation Problem for the Wounded ...** The Recent Heavy Rush of Work at Cambridge. | 177 |
| **Round the World ... ... ... ... ... ...** Fellow-Workers and the Roll of Honour. | 178 |
| **The Birmingham Scheme of Classified Institutions ... ... ... ... ... ... ...** What it Does, and Where it Fails. | 179 |
| **Metaphysics and Mental Disease ... ...** | 180 |
| **Duty-Free Rectified Spirit in Hospitals ...** Objections Answered and New Points Urged. | 181 |
| **From the Patient's Point of View ... ...** A Nurse's Experience as a Patient. | 182 |
| **The Editor's Letter-Box ... ... ... ...** Early-closing Day at Chemists' Shops. | 184 |
| **Medical Appointments ... ... ... ...** | 184 |
| **Gifts and Bequests ... ... ... ... ...** | 184 |
| **The Book Market ... ... ... ... ...** | 184 |
| **The Institutional Worker .. ... (Suppl.)** Answer to October Questions. Nut Butter in Institutions. Questions for November. Questions Invited. The Sick and in Need. War Matters. | 1 |

